# Developing Conflict Resolution Strategies and Building Resilient Midwifery Students: Protocol for a Mixed Methods Research Study

**DOI:** 10.2196/35558

**Published:** 2022-02-18

**Authors:** Naomi Simpson, Mary Steen, Rachael Vernon, Annette Briley, Dianne Wepa

**Affiliations:** 1 Department of Clinical and Health Sciences University of South Australia Adelaide Australia; 2 Department of Nursing, Midwifery and Health Faculty of Health and Life Sciences Northumbria University Newcastle United Kingdom; 3 College of Nursing and Health Sciences Flinders University Bedford Park Australia

**Keywords:** midwifery students, workplace, bullying, violence, conflict, abuse, resilience

## Abstract

**Background:**

Workplace bullying and violence (WBV) are well-documented issues in the midwifery profession. Negative workplace culture, conflict, and bullying are the most common forms of workplace violence experienced by midwives. Workplace violence increases the risk of midwives experiencing burnout, compassion fatigue, psychological trauma, poor mental health, absenteeism, loss of passion for the midwifery profession, job dissatisfaction, and poor job retention. Midwifery students describe workplace violence in the form of physical, emotional, or verbal abuse, and bullying. Therefore, there is a justification to develop conflict resolution strategies and resilience in midwifery students prior to graduation.

**Objective:**

Our aim is to develop and facilitate a bespoke education program for South Australian midwifery students to enable them to develop skills in conflict resolution, build resilience, and identify self-care strategies.

**Methods:**

This study will undertake a preparatory phase summarizing the body of literature on midwifery students’ knowledge, understanding, and experiences of WBV. Following this, a 3-phase sequential mixed methods research design study will be undertaken. In Phase 1, quantitative data will be collected via a semistructured questionnaire and a validated conflict measurement tool, before and after attending an education workshop, and will be analyzed using descriptive and inferential statistics. Results from Phase 1 will inform and guide the development of an interview schedule for Phase 2. In Phase 2, qualitative data will be gathered by facilitating one-to-one interviews and a thematic analysis will be undertaken to gain a deeper understanding of midwifery students’ experiences of WBV. In Phase 3, data integration using triangulation will be undertaken and meta-inferences will be developed via the integration of results and findings from Phases 1 and 2.

**Results:**

The preparatory phase will commence in October 2021. Phase 1 will commence in 2022 with analysis of pre- and posteducation results anticipated to be completed by December 2022. Phase 2 will be developed from findings of the preparatory phase and results of Phase 1. An interpretation of verbatim interview transcripts is estimated to be undertaken by April 2023. Phase 3 of the study is expected to commence in May 2023, and this will involve the analysis of collective evidence gathered from Phases 1 and 2. The anticipated completion date for the study is December 2023.

**Conclusions:**

The outcomes of this research will provide insights into the prevalence and impact of WBV experienced by midwifery students. The findings of the research will report on levels of knowledge, skills, and confidence, and will assess the impact of a bespoke conflict resolution and resilience education workshop for midwifery students in managing WBV.

**International Registered Report Identifier (IRRID):**

PRR1-10.2196/35558

## Introduction

### Background

Workplace bullying and violence (WBV) are well-documented concerns in the midwifery profession [1-3], with midwifery students reporting experiences of WBV in clinical settings, thus identifying the need to develop conflict resolution strategies and resilience in undergraduate education programs [3-6]. Workplace bullying is defined as “seek to harm, intimidate or coerce someone seen as vulnerable” [7]. Bullying is a repeated pattern of violence over time, which brings harm to the impacted individual(s) [8].

Workplace violence is an act or threat of physical abuse, harassment, intimidation, or other threatening disruptive behavior that occurs in the workplace [9]. Workplace violence can be caused by an individual or a group of people. Midwifery students describe experiencing workplace violence in the form of physical, emotional, or verbal abuse, and bullying [10]. Workplace violence is emotionally, psychologically, and spiritually damaging and can have long-lasting effects on the impacted individuals. The negative consequences associated with WBV include absenteeism, stress, fatigue, psychological trauma, poor mental health, job dissatisfaction, and poor job retention [5,10-13].

Workplace culture is defined as the beliefs, values, assumptions, attitudes, behavior, customs, and social interactions of staff in a particular workplace [14,15]. Workplace culture can affect the professional practice of a midwife, the way midwives interact with women, and impact upon the learning ability of midwives [14]. Toxic workplace culture promotes negative behavior, increasing the risk of WBV [16]. As a result of negative workplace culture, midwives experience burnout, job dissatisfaction, and attrition [14].

The midwifery profession is a female-dominated workforce that provides woman-centered maternity care by working in partnership with the woman [17]. The Nursing and Midwifery Board of Australia (NMBA) states that midwives should engage in respectful partnerships with the woman as well as other professionals and colleagues [18]. Despite this professional requirement, evidence has shown that midwives commonly bully other midwives, showing disrespect and a lack of compassion for each other in the workplace. This is particularly true for midwifery students and graduate midwives who are more vulnerable [10].

It has been reported that all new graduate midwives must adapt and learn quickly when becoming responsible for the care of women during the perinatal period [19,20]. The pressure of adjusting to shift work increases the risk of stress and fatigue for graduate midwives [21]. It has been highlighted that negative workplace culture, conflict, and bullying from colleagues and senior staff are the most common forms of workplace violence experienced by graduate midwives [11,13,22-24]. Additionally, WBV increases the risk of graduate midwives experiencing burnout, compassion fatigue, poor mental health, absenteeism, loss of passion for the midwifery profession, job dissatisfaction, and attrition [5,10-13].

Graduate midwives have reported experiences of coercion as a form of bullying, from senior midwives and doctors, resulting in them feeling forced into pressuring women to undergo procedures, including vaginal examinations and artificial rupture of membranes [25,26]. Coercing women is in opposition with the holistic approach ensconced in woman-centered care, thus leading to a moral dilemma for the midwife, which in turn may compromise care provision [18,25,27-30]. While this behavior is not a reflection of the midwife, it is a result of the culture within the midwifery profession, which fosters ethically and morally questionable care, due to institutional constraints [30]. Kumar-Hazard [31] has suggested that asking midwives to work within their employment contract, and provide woman-centered care, is an untenable situation.

Midwives have experienced coercion from hierarchical relationships (senior midwives, doctors, and management) to use wording that pressures the birthing woman to undergo unnecessary investigations and interventions, which impacts upon the midwife’s ability to provide woman-centered care and her professional autonomy [31]. This coercion is presented under the guise of protecting the baby’s well-being [31-33]. Coercion often results from a misuse of power differentials within the workplace and may lead to increased mental distress, moral dilemma, psychological trauma, job dissatisfaction, and loss of passion for the midwifery profession. This is particularly the case for graduate midwives who commonly feel ill equipped to challenge the system, or those they work with [13,29,34]. Contradictions between woman-centered care and institutional constraints are a significant reason why midwives choose to leave the midwifery profession [30]. Davies and Coldridge [30] have highlighted that witnessing poor practice was a central predictor for midwifery students losing their passion for the midwifery profession and was associated with high attrition rates, justifying the need to develop conflict resolution strategies and build resilience.

In a predominantly medicalized system, where midwives are losing their voices, midwifery students need to have skills in conflict resolution and to be resilient [35], as stated by Warland et al [36] “to safely manage their own behaviour and the behaviour of others”. Conflict resolution skills and resilience may enable student and graduate midwives to advocate for women in their care and provide holistic care, while coping with trauma and adversity [35,37-40]. Unresolved conflict may impact workplace relationships and increase stress, which impacts upon the mental health of midwives and affects their ability to provide woman-centered care [5,41,42].

Building resilience has been acknowledged as an empowering concept that enables midwives to endure or recover quickly from challenging situations [7,40,43,44]. Clohessy et al [43] describe the benefits of resilience to include an “effective coping or adaptive capacity and a positive mental health status,” which enables midwifery students to overcome stress and adversity. Richardson [45] and Hunter and Warren [46] suggest that resilience can be learned or developed. This idea was further explored by Taylor and Reyes [47] who concurred that resilience could be learned or enhanced through education strategies. Clohessy et al [43] have described how midwifery students reported using strategies for resilience, such as confidence, optimism, reflection, and social supports, to manage exposure to adversity.

Despite acknowledgement of the ongoing nature of WBV in the midwifery profession [1-3], there has been minimal change over the last 35 years in Australia [2]. One potential solution to the challenges identified is the development of a bespoke educational workshop within the Bachelor of Midwifery degree, which may support midwifery students to develop skills in conflict resolution, build resilience, and identify self-care strategies, preparing them to manage conflict when they enter the workforce. As a result, burnout, attrition, and the loss of passion for the midwifery profession may be reduced.

### Aim

The aim of this study is to develop and facilitate a bespoke education program for South Australian midwifery students, to enable them to develop skills in conflict resolution, build resilience, and identify self-care strategies.

### Objectives

#### Preparatory Phase

In this phase, we will explore the body of literature relating to midwifery students’ knowledge and experiences of conflict in the workplace.

#### Phase 1

The objectives of this phase are as follows:

- To develop a bespoke education workshop in conflict resolution skills and build resilience in midwifery students.
- To facilitate and evaluate the impact of an education workshop on developing conflict resolution skills and resilience for midwifery students.
- To assess second-year midwifery students’ knowledge and skills to manage conflict in the workplace.
- To explore midwifery students’ ability to be resilient after attending an education workshop.
- To further explore midwifery students’ levels of confidence in addressing WBV after attending an education workshop.

#### Phase 2

The objectives of this phase are as follows:

- To gain a deeper understanding of midwifery students’ skills and ability to manage conflict in the workplace while on placement or providing continuity of care.
- To explore midwifery students’ views and experiences of using conflict resolution strategies.

#### Phase 3

The objectives of this phase are as follows:

- To triangulate and integrate data to strengthen the results and findings.
- To provide evidence and draw conclusions to answer primary and secondary research questions and the aim and objectives of this mixed method study.

#### Primary Research Question

1. What impact will a bespoke educational workshop to develop conflict resolution strategies and resilience have upon a population of midwifery students when facilitated during the Bachelor of Midwifery program?

#### Secondary Research Questions

1. What knowledge and skills do midwifery students’ have to manage conflict in the workplace, before receiving education?
2. What impact will education have upon midwifery students to develop conflict resolution skills?
3. What impact will education have upon midwifery students to develop and build resilience to manage WBV?
4. What are the views and experiences of midwifery students after receiving education to develop conflict resolution strategies to manage conflict in the workplace?
5. What are the views and experiences of midwifery students after receiving education to develop and build resilience to manage conflict in the workplace?

## Methods

### Study Design

The research study will utilize a sequential explanatory mixed method design as suggested by Fielding [48] and Fetter et al [49]. A 3-phase design will be undertaken to gather both quantitative and qualitative data in a sequential manner, followed by meta-inferences in the final phase. This study design will enable researchers to explore students’ views and experiences of WBV before and after attending an educational workshop.

The preparatory phase will review literature and provide evidence to inform the researchers as to what is currently known about knowledge, skills, views, and experiences of midwifery students regarding WBV. The preparatory phase will provide an evidence base for exploring the research problem, which will assist in the development of pre- and postquestionnaires, the content of an educational workshop, and development of an interview schedule. Phase 1 will involve collecting quantitative data, and SPSS (IBM, Inc.) will be used to perform descriptive and inferential statistical analyses. A pre-education questionnaire will be developed and include validated conflict scales to evaluate midwifery students’ knowledge, skills, and confidence regarding WBV [50]. A posteducation questionnaire will re-evaluate midwifery student’s knowledge, skills, and confidence of WBV, to investigate the impact of the conflict resolution and resilience educational workshop. Phase 1 will investigate and provide a general overview of the results related to the research topic. In Phase 2, an interview schedule of semistructured questions will be developed from the evidence generated in the preparatory phase and Phase 1, thus adopting a sequential design for the research. Phase 2 involves the collection of qualitative (written text) data gathered from one-to-one interviews, which will be transcribed verbatim and thematically analyzed. Phase 2 will explore and explain in more depth the results from Phase 1. Phase 3 will involve the merging of Phase 1 results and Phase 2 findings, and the collective data will be triangulated and then integrated to determine meta-inferences from quantitative and qualitative data, to draw conclusions and understanding of the research questions [51].

### Mixed Methods

Using a mixed methods research design that involves the analysis of quantitative and qualitative data provides stronger evidence and strengthens conclusions drawn [52], which neither quantitative nor qualitative research cannot achieve alone [53]. This study design ensures that the research project is robust and supports the broad understandings and conclusions of the research phenomena [54]. Mixed methods research allows flexibility of findings in the research, using multiple methods, worldviews, representation of assumptions, data collection, and analysis [51]. The flexibility of mixed methods design enhances the approach undertaken to address the research phenomena [51]. The strength of the explanatory sequential framework that will be utilized for this proposed study is that the research phases build upon each other [52]. Quantitative data will be analyzed in the first phase, with qualitative findings being subsequently analyzed to help explain the quantitative results, giving strength and validity to the study. As a result, the research is more than an evaluation, it is an investigation and exploration of midwifery students’ experiences of WBV.

### Conceptual Framework

Pragmatism is recognized as a world view that accepts several realities and supports practicality when addressing research questions. Kaushik and Walsh [55] have defined pragmatism as “a way of thinking about and making sense of the complexities of the real world”. This concept reflects both biased and unbiased perspectives. The pragmatic concept utilizes a philosophical methodology that draws on utilizing *what works* from different aspects, setting priorities for the research topic/problem and questions, gathering both objective and subjective data [56]. Pragmatism uses an integrative philosophy, which combines both quantitative and qualitative research, without restrictive methodological directions [56,57].

The research study will use pragmatism as the underlying philosophy as this concept informs both quantitative and qualitative data collection [52]. Pragmatism is often associated with mixed methods research as the focus is guided by the research questions and the consequences of the research, as opposed to the methods [57]. Pragmatism provides an experience-based, action-orientated framework that can be utilized in a practical setting [58].

### Setting

The research will be completed in metropolitan South Australia. Participants will be sought from 2 Universities in South Australia that offer the Bachelor of Midwifery degree.

### Sampling

A purposive sample of second-year midwifery students completing the Bachelor of Midwifery degree in South Australia will attend an educational workshop as part of their curriculum studies, and be invited to participate in the research study.

### Participants

The study will recruit second-year Bachelor of Midwifery students from 2 universities in South Australia. In Phase 1, all second-year Bachelor of Midwifery students will be eligible to participate in the research, and those providing written informed consent to participate will be included. In Phase 2 of the research, the study will consider any second-year midwifery students who have personally witnessed or experienced conflict in a clinical setting, while on placement or continuity of care experiences, who provide written informed consent to participate in this study.

### Phase 1 Participants

#### Inclusion Criteria

- Midwifery students enrolled in the second year of Bachelor of Midwifery degree in a University in South Australia offering the Bachelor of Midwifery degree.
- Midwifery students giving verbal and written informed consent.

#### Exclusion Criteria

- Midwifery students not enrolled in the second year of Bachelor of Midwifery degree.
- Midwifery students undertaking the second year of Bachelor of Midwifery degree outside of South Australia.

### Phase 2 Participants

#### Inclusion Criteria

- Midwifery students enrolled in the second year of Bachelor of Midwifery degree in a University in South Australia who have personally witnessed or experienced conflict in a clinical setting, while undertaking placement or continuity of care experiences.
- Midwifery students giving verbal and written informed consent.

#### Exclusion Criteria

- Midwifery students not enrolled in the second year of Bachelor of Midwifery degree.
- Midwifery students undertaking the second year of Bachelor of Midwifery degree outside of South Australia.

### Recruitment

#### Phase 1

All second-year Bachelor of Midwifery students will be required to attend the bespoke education workshop on conflict resolution strategies and building resilience as part of the Bachelor of Midwifery degree. Second-year midwifery students from the respective universities will be provided with information about the research project by a university staff member not associated with the research. Following the delivery of this information, second-year midwifery students will be invited to participate in the research. Midwifery students who give verbal and written informed consent will be invited to complete the pre- and posteducation workshop questionnaires. Student participants will also be informed of the potential to be involved in posteducation workshop interviews to follow-up questionnaire responses, where eligible.

#### Phase 2

A purposive sample of eligible second-year midwifery students from Phase 1, who have witnessed or experienced conflict in the workplace, will be invited by the primary researcher (NS) to participate in a posteducation workshop follow-up interview. The interview will focus on exploring student participants’ views to gain a deeper understanding and insights into their experiences of WBV. The primary researcher will connect with eligible students via their university email address and will arrange either one-to-one, telephone, or Zoom interview with students agreeing to participate in Phase 2 of the research. Additional written informed consent will be obtained prior to participation in this phase of the study.

### Bespoke Educational Workshop

The Start Treating Others Positively (STOP) model (Multimedia Appendix 1) will be incorporated into the design and development of an educational workshop for this research study [59]. The STOP Model originated in 1989 and was introduced to health care settings in the United Kingdom in 2001 [59]. The STOP model approach was adopted, and workshops were developed, to build conflict resolution strategies as part of an abusive behavior management program for adults [59]. STOP is a strengths-based model that utilizes a positive approach encouraging good decision making for the future, rather than focusing on unchangeable actions of the past [4], thus supporting personal behavior change [59]. The key steps of the STOP model include

*STOP*: Stop and see what is happening. Don’t just react!

*THINK*: What is important here? What could be the threat?

*OBSERVE*: Calmly work out the problem.

*PROCEED*: Take time out? Be assertive.

The STOP model was modified to be included in antenatal education, with the aim of enabling and empowering expecting parents to manage their emotions and behaviors, with the overarching goal of preventing relationship conflict escalating to abuse [60]. Antenatal participants proposed that the STOP model be included routinely in antenatal education, suggesting that “introducing the tools and techniques at the beginning of the parenthood journey might break the cycle with the parents before the next generation of children encounter it.” Participants further recommended including STOP in formal school education [60].

STOP was introduced to midwifery students attending the University of Chester in the UK in 2010 [4]. The content was adapted from the original design, to meet the needs of midwifery students. Steen [4] utilized a posteducation assessment, to evaluate midwifery students’ knowledge and skills after attending the STOP workshop. The adapted STOP model underpinned the educational workshop to enable midwifery students to develop conflict resolution skills and build resilience in the event they experienced WBV. Students who participated in STOP education and training demonstrated new insights into how to manage workplace conflict and reported utilizing STOP strategies throughout the rest of their studies, after graduating and in personal circumstances [4].

Therefore, the STOP model will be utilized as a framework to guide the development and facilitation of an educational workshop for midwifery students in South Australia, to develop conflict resolution strategies, and to build resilience. As negative human behavior involving bullying and violence is similar within Western societies [61-63], it is acceptable to use the STOP model framework that was developed in the UK and adapt this approach to meet the needs of midwifery students in South Australia.

### Ethical Considerations

#### Approval

Ethical approval has been obtained from the University of South Australia Human Research Ethics Committee (Protocol Number 204063). Approval has been sought from Flinders University. Ethical considerations (ie, informed consent, anonymity and confidentiality, voluntary nature of participation and withdrawal) have been addressed at all stages of the research project study design. Verbal and written consent will be obtained from all participants.

#### Consent

Midwifery students attending the bespoke educational workshop will be provided with a participant information sheet (PIS) explaining the research being undertaken. The PIS includes information pertaining to the study, that is, aim, objectives, potential outcomes, content of workshop, evaluation of workshop, and Phase 2 interviews. The PIS will include details on participation within the study including the benefits and risks, participant confidentiality, and the support strategies put in place to address any distress resulting from the study. Participants will be provided with contact details for the research team in the event that they have further questions they would like answered, prior to making an informed decision and thus providing informed consent to participate in the research.

#### Participants Safety and Withdrawal

Participation in the study is voluntary. Participants have the right to withdraw from the study at any time. Withdrawal forms will be uploaded onto the REDCap web platform for midwifery students to access if required [64]. REDCap is a safe and secure web platform for developing and managing online surveys and databases [64]. Data collection up to the point of withdrawal will be included within the data analysis. No further data will be collected following withdrawal of participation from the research. Withdrawing from the study will not affect midwifery students’ relationship with the research team or their respective university.

#### Confidentiality

A purposive sample of second-year South Australia midwifery students will be invited to participate in the research study by an academic staff member from their respective university who is not involved in the research study. Midwifery students will be provided with participant information for the study during recruitment, as part of the informed consent process. The pre- and posteducation workshop questionnaires, validated conflict scale, and interview responses will be deidentified with codes when collating data and prior to publishing results and findings, to protect the participants and ensure anonymity within the research. Midwifery students will be able to access the research information and questionnaires through the REDCap web platform from any device that has internet access [64]. The information and questionnaires will also be provided in hard copy form if any participant prefers. Hard copies of completed questionnaires will be stored in a locked filing cabinet, in a locked office at the University of South Australia for the entirety of the research project to protect the confidentiality of participants.

At all times, participants personal information will remain confidential within the research team. No information will be released by researchers that may lead to midwifery students’ identification unless required by relevant legislation.

#### Data Management

University of South Australia data management policy and guidance will be adhered to regarding the appropriate data storage of, access to, and destruction of information/data gathered during the undertaking of the research project [65].

Research data will be deidentified with predetermined codes. A separate document with midwifery students’ university email address (identifying factor) and their particular identifying code will be password protected and stored as a file on a personal computer, USB, and hard drive. Only the research team will have access to identifying data.

All digital audio files and data will be coded, archived, and stored in a password-protected file on the university server. To reduce risk of file corruption or loss, files will be stored in at least two locations. All coded hard copy and transcribed audio data (questionnaires and interviews) will be stored in a locked filing cabinet in a secure facility with access restricted to the research team, for a minimum of 5 years according to the university data management and policy guideline for general research. After such time, secure data destruction will take place. These measures will be taken to ensure security of information from misuse, loss, or unauthorized access while stored during the research project and on completion.

### Procedure

#### Phase 1: Investigation

##### Overview

In Phase 1 of the research, participants knowledge and skills regarding WBV will be assessed prior to the facilitation of a 3-hour bespoke educational workshop to develop conflict resolution strategies and build resilience. Following the educational workshop, midwifery students’ knowledge and skills relating to WBV and the effectiveness of the workshop will be assessed twice. Assessment will take place immediately after workshop and then again 8-12 weeks later when students have had an opportunity to complete further clinical experiences.

##### Data Collection Tools

These include (1) a piloted pre- and posteducation questionnaire and (2) a validated conflict assessment scale/tool [50].

Pre- and postworkshop questionnaires will be developed by the research team, led by key concepts within the STOP model [4]. These questionnaires will be piloted in a group of approximately 5 midwifery students who will have the opportunity to comment and provide feedback on the questionnaire. The questionnaires will gather data regarding the knowledge and skills of midwifery students relating to WBV while undertaking clinical experiences, as part of the requirement for the Bachelor of Midwifery degree. These data will be mainly collected through 5-point ordinal Likert scales as a preferred measurement for health research [66]. Midwifery student participants will be provided with a series of questions and statements and have an opportunity to expand on their answers in some sections of the questionnaire.

A validated conflict measurement tool [50] will be adapted by the research team for use in this study. This tool will incorporate 5-point ordinal Likert scales that are designed to assess negative behaviors in the workplace, associated with workplace bullying.

##### Data Analysis

Five-point ordinal scales will be included in pre- and postquestionnaires to measure midwifery students’ responses. Descriptive and inferential statistics using SPSS version 26 (IBM) will be used to measure responses gathered by the online questionnaires developed in the REDCap web platform [64]. SPSS is an interactive statistical analysis program that analyses data from most files. REDCap files can be directly imported into and with SPSS software. Data will be presented visually in charts and as graphs to help report results.

#### Phase 2: Exploratory

##### Interviews

Eligible second-year midwifery students will be invited to participate in posteducation workshop interviews, to discuss their views and experiences of WBV and what impact the educational workshop has had upon them to manage WBV.

Semistructured interviews will be conducted using open-ended questions so that the researchers can explore midwifery students’ views and experiences of conflict in the workplace [67,68]. Semistructured interviews will help to facilitate in-depth discussion and guide the exploration of research questions [67].

An interview guide will be developed and comprise approximately 10 main questions, and some further subquestions to help prompt the student participant to continue discussing their views and experiences [67,68]. The interview questions will be piloted before the research team proceeds with Phase 2 of the research.

The research questions will be asked in a flexible and friendly manner so that participants will feel comfortable and safe to participate fully in the interview [68]. At the end of an interview, the researcher will summarize the main findings of the interview to the participant, to cross-check if they have an accurate representation of their views and experiences [69]. At this stage, midwifery students may choose to clarify, add, or omit anything. Interviews will be recorded, transcribed verbatim, and a thematic analysis will be undertaken.

##### Data Saturation

Data saturation will be reached when no new themes emerge from the research [69]. Hennink et al’s [70] framework will be used as a guide to reach data saturation. It is anticipated that a minimum of 10-12 interviews will be conducted to gain an insight into the views and experiences of midwifery students, and their ability to manage WBV in the workplace when exposed to this phenomenon.

##### Data Analysis of Interviews

Recordings of interviews will be transcribed verbatim using a transcription service. Once data have been transcribed, digital files of recorded interviews will be kept on a computer, password protected, to keep the research data confidential and limited to the research team. Data files will be backed up on the university server frequently to reduce risk of complete loss.

A constant comparative method will be utilized, to compare and analyze data after each interview as recommended by Richards and Hemphill [71]. Braun and Clarke’s [72] reflexive thematic analysis framework will be utilized to generate themes. Researchers will be guided by 6 steps as first described by Braun and Clarke [72]. These steps include the following:

1. Familiarizing: focusses on immersing oneself in the data and noting initial ideas;

2. Generating: involves developing codes from features found within the data;

3. Searching: provides links between established codes and themes uncovered within the data;

4. Reviewing: appraises themes to ensure they work with the data captured;

5. Defining: provides clarity of each theme and the overall story and generates clear definitions;

6. Reporting: synthesizes the final analysis of the extracts, referring back to the research question and literature (Multimedia Appendix 2).

#### Phase 3: Triangulation and Meta-inferences

Quantitative and qualitative data will be triangulated, integrated, and meta-inferences of the collective data will be developed. The explanatory sequential mixed methods design will be guided by the following principles recommended by Tashakkori and Teddlie [51].

1. *Triangulation and corroboration* of results/findings to increase the external validity of results.
2. *Complementary*: Seek to add clarification, elaboration, and enhancement of the results from Phase 1, with the findings from Phase 2, and have 1 summary (joint display) of findings built upon the other, contextualizing information and adding a macro complementary picture of the research phenomena.
3. The results of Phase 1 will be used to develop Phase 2 data collection questions and gathering of the findings.
4. *Exploratory*: Phase 2 (qualitative) will enable the exploration of themes or new perceptions and possible re-design of some more in-depth research questions or explore views/experiences with the outcomes and investigate further the information gathered.
5. *Comparison*: Phase 1 (quantitative) and Phase 2 (qualitative) methods will be used to compare and identify inconsistencies of the research topic.
6. *Expansion*: Combined data will be used to extend details about the horizon of research and widen the understanding of the topic.

## Results

This research will provide both quantitative and qualitative data to determine midwifery students’ knowledge, and skills relating to WBV in a clinical setting, while undertaking their Bachelor of Midwifery degree. It is anticipated that education will have an impact on how midwifery students will manage WBV in the workplace by developing some conflict resolution skills and building resilience. This research will provide valuable insights into the views and experiences of midwifery students regarding WBV. Integrated data will help draw conclusions and recommend future implications for midwifery student education.

This study is expected to conclude in December 2023.

## Discussion

### Importance of This Research

Evidence has shown that there is a culture of bullying and abuse within the midwifery profession. Several studies have reported that midwives, graduate midwives, and of significant note, midwifery students (who are more vulnerable) have witnessed or experienced WBV [10,73-75]. It has been highlighted that midwifery students feel a lack of preparation in managing WBV [11], which may result in midwifery students emulating poor behavior of midwives and midwifery leaders [5,76]. Hogan et al [3], Steen [4], and Capper et al [11] suggest that midwifery students require additional pathways to address WBV; however, it was acknowledged by Capper et al [11] that there has been no intervention study attempted to date.

To address this deficit, the STOP model [4] will be used as a framework to underpin and guide development and facilitation of a bespoke educational workshop to develop conflict resolution strategies and build resilience for this research. It is anticipated that this research study will address gaps in the literature and provide evidence to confirm or refute any benefits from providing education and training for conflict resolution strategies and building resilience. Building resilience in midwifery students may improve their self-confidence to deal with trauma and adversity, resulting in improved sustainability for the midwifery profession [38,46].

Therefore, developing a bespoke educational workshop that utilizes a validated conflict resolution model [4] may justify the adoption of implementing conflict resolution strategies and building resilience skills as essential curriculum content within a Bachelor of Midwifery degree program to meet the learning needs of midwifery students in South Australia.

### Conclusion

There appears to be limited research to inform the development of personal resilience and conflict resolution strategies within Bachelor of Midwifery degree programs. The results and findings from this sequential mixed methods study will be triangulated, and data integration will develop meta-inferences that will strengthen the conclusions drawn. Outcomes from the research will elucidate the experience, potential impact, and prevalence of WBV by midwifery students in South Australia. It is anticipated that the findings will inform recommendations for future midwifery education programs and may include the implementation of conflict resolution and resilience workshops within Bachelor of Midwifery degree curricula.

## Appendix

Multimedia Appendix 1 The Start Treating Others Positively model.

Multimedia Appendix 2 Braun and Clarke’s 6-stage framework (2006, page 87).

Multimedia Appendix 3 Peer-reviewer report from a committee prepared by the University of South Australia, Clinical and Health Sciences.

## Abbreviations

NMBA: Nursing and Midwifery Board of Australia
PIS: participant information sheet
STOP: Start Treating Others Positively
WBV: workplace bullying and violence
