# Supplementary figures and images for: Developing Conflict Resolution Strategies and Building Resilient Midwifery Students: Protocol for a Mixed Methods Research Study
Source: JMIR Res Protoc. 2022 Feb 18;11(2):e35558. doi: 10.2196/35558 (PMC8900901; doi:10.2196/35558)

Multimedia Appendix 1. The Start Treating Others Positively model.


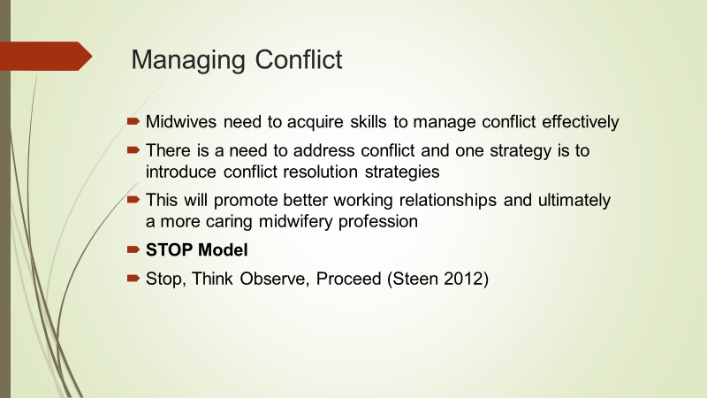


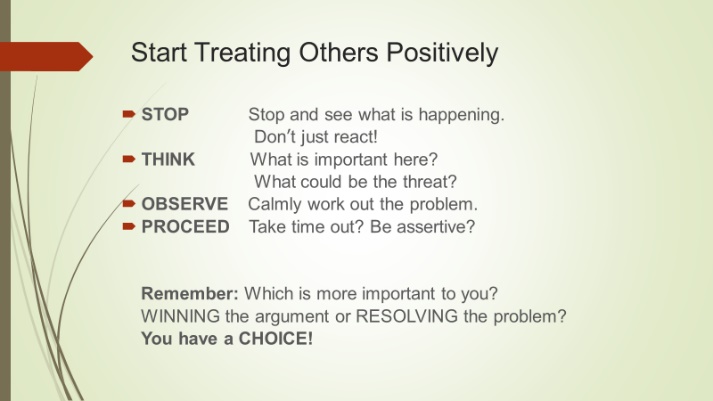


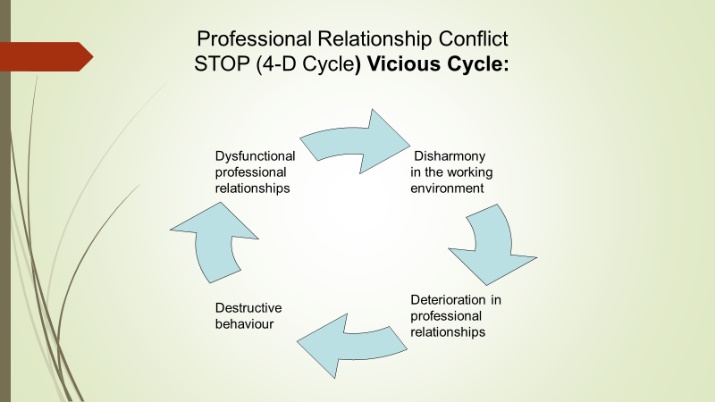


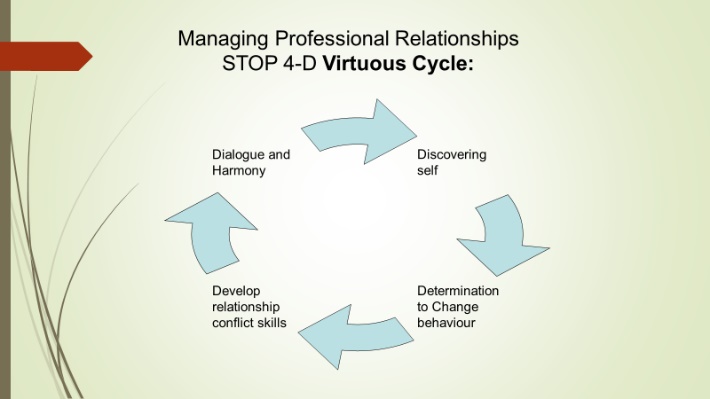

Supplement: Multimedia Appendix 1 [file resprot_v11i2e35558_app1.docx]
