# Supplementary material for: Developing Conflict Resolution Strategies and Building Resilient Midwifery Students: Protocol for a Mixed Methods Research Study
Source: JMIR Res Protoc. 2022 Feb 18;11(2):e35558. doi: 10.2196/35558 (PMC8900901; doi:10.2196/35558)
Supplement: Multimedia Appendix 2 [file resprot_v11i2e35558_app2.docx]

Multimedia Appendix 2. Braun and Clarke’s 6-stage framework (2006, page 87).

| **Phase** | | **Description of process** |
| --- | --- | --- |
| 1 | Familiarising yourself with your data | Transcribing data (if necessary), reading and re-reading the data, noting down initial ideas. |
| 2 | Generating intial codes | Coding interesting features of the data in a systematic fashion across the entire data set, collating data relevant to each code. |
| 3 | Searching for themes | Collating codes into potential themes, gathering all data relevant to each potential theme |
| 4 | Reviewing the themes | Checking if the themes work in relation to the coded extracts (Level 1) and the entire data set (Level 2), generating a thematic ‘map’ of the analysis. |
| 5 | Defining and naming themes | Ongoing analysis to refine the specifics of each theme, and the overall story the analysis tells, generating clear definitions and names for each theme. |
| 6 | Producing the report | The final opportunity for analysis. Selection of vivid, compelling extract examples, final analysis of selected extracts, relating back of the analysis to the research question and literature, producing a scholarly report of the analysis. |
