# Supplementary material for: Developing Conflict Resolution Strategies and Building Resilient Midwifery Students: Protocol for a Mixed Methods Research Study
Source: JMIR Res Protoc. 2022 Feb 18;11(2):e35558. doi: 10.2196/35558 (PMC8900901; doi:10.2196/35558)
Supplement: Multimedia Appendix 3 [file resprot_v11i2e35558_app3.pdf]

PhD Feedback from examiners

Examiner	Feedback from examiners	Primary researcher comments
1= JF		
2 = MC		
	**TOPIC**	
1	More detail to be provided for theoretical framework	• Expanded the theoretical framework. See p. 14, 15, 16, 17.
	**BACKGROUND**	
2	Need to address root problem – that bullying, and violence are not acceptable however they come.	Thank you for your comments and feedback. Unfortunately, on discussion with my supervisors, we feel that looking at the highly complex root cause of workplace violence and bullying in Midwifery is out of the scope of the PhD. We feel that this is an important discussion that requires investigation, however that it is better suited to post graduate work with possible funding.
	**LITERATURE REVIEW**	
2	Further discussion needed around the meaning of workplace culture, bullying and violence and more specifically, the interplay between them in the midwifery profession. Distinct differences that need to be articulated more clearly.	• Expanded discussion of workplace culture, bullying and violence. See p. 3.
2	Mention of trauma needs to be introduced earlier and linked more strongly to what is trying to be achieved by the research.	• See p. 3, 4, 7.
1	More detail could be provided to justify your approach – impact of WBV and factors that might contribute including issues such as hierarchical environments	• Provided background to show importance of research topic. See p. 3, 4, 5, 6, 7, 8, 9.<br>• Acknowledged that there is research to show that pathways need to be investigated to address WBV. See p.7, 10<br>• Acknowledged that there has been no intervention study to date in midwifery addressing WBV. See p. 10<br>• Acknowledged that there are g,s in knowledge. See p.10

		• Described the validation of using STOP model. See p.9, 10
1	A stronger link required to justify the proposed intervention and approach to this issue	• Background included. See p.10<br>• Need for education intervention highlighted. See p.10<br>• STOP model strengthened. See p.9<br>• Gaps in knowledge acknowledged. See p.10<br>•
1	More detail required to explain STOP model. Include a summary of how the model was delivered ie. Timeframe of intervention, resources (workshop plan and tools previously used in evaluation). Discussion on how the STOP model will be adapted to Australian landscape	• Evaluated workshop. See p.9<br>• STOP model will be used as a framework and content will be adapted to meet the needs of SA third year/ final year midwifery students. See p.9
1	Include Warland et al. 2014 paper on assertiveness training and papers by Chlohessy et al. 2019 on resilience.	• Included reference to Clohessy, McKellar & Fleet (2019) See p. 5, 7.<br>• Included reference to Warland, McKellar & Diaz (2014) See p. 5, 8.
	**PROPOSAL**	
1	Additional details needed in some sections	• Completed as per intext comments in proposal.
	**RESEARCH METHOD**	
1	More detail required regarding participants – inclusion / exclusion eg. SA midwifery students. ? Graduate midwives?	• Inclusion / Exclusion criteria completed. See p. 13<br>• 3rd year / final year midwifery students added to sample. See p.13
1	How many participants are you hoping to recruit ie. Interviews 10	• Numbers approximated from university information on sample sizes from respective Universities of students eligible to attend workshop. See p. 13
1	Identification of how sample size was identified	• Purposive, convenience sample of 3rd year / final year SA midwifery students. See p. p.13
1	No data collection tools were discussed	• Validated conflict tool described. See p. 18,19.

		• Pilot study will be completed to assess the questionnaire. Approximately 5 midwifery students will be invited to participate in the pilot study, in which the students can provide comments and give feedback on the questionnaire. See p. 19.
1	Expand of figures	• Have slightly expanded discussion of figures and included labels as already over 20 pages. See p. 15, 16.
1	Discuss type of literature review – ie. Integrated review	• Integrated literature review. See p. 11, 13, 15 & 16
1	How does this preparatory phase inform the development of the workshop?	• The literature review will serve as the basis for knowledge on WBV for midwifery students and will have the capacity to direct the development of questionnaires (Phase 1), the content of the workshop and the development of interview questions (Phase 2). See p. 15
1	Consider – Interview to inform development of workshop – sequentially?	• We considered this, however I have chosen to undertake an Explanatory Sequential design, so that the findings from the interviews in Phase 2, help to explain the results of data collected from Phase 1.
	**ETHICS**	
1	More detail needed on how you propose to address ethical considerations such as recruitment strategies	• See p. 21, 22.<br>• NHMRC Australian code for responsible conduct & UniSA Framework for responsible conduct were consulted<br>• Convenience sample for workshop<br>• Participants invited to participate in study<br>• Participants provided will be provided with research information in the form of a participant information sheet (Appendix), so that they can make informed decisions<br>• Consent form (Appendix)<br>• Withdrawal form (Appendix)<br>• De-identified codes to ensure confidentiality<br>• Students to access REDCap research platform from any device with internet access

		• Participants will have a support strategy, as outlined in the participant information sheet, to minimise harm in the event that the themes discussed in questionnaires, interviews or workshop cause distress<br>• Ethical approval to be sought from the Human Research Ethics Committee at UniSA prior to the study commencing.
1	Information required re: data management plan	• Data management plan discussed. See p. 20, 21
	**REFERENCING**	
1	UniSA Harvard referencing to include page numbers?	• Apologies, I didn’t have time to complete this prior to oral defence, but will update all references to reflect this.
